# A data model and file format to represent and store high frequency energy monitoring and disaggregation datasets

**DOI:** 10.1038/s41598-022-14517-y

**Published:** 2022-06-18

**Authors:** Lucas Pereira, Nuno Velosa, Manuel Pereira

**Affiliations:** 1 ITI, LARSyS, Técnico Lisboa, 1049-001 Lisbon, Portugal; 2 Universidade da Madeira, 9020-105 Funchal, Portugal

**Keywords:** Computer science, Scientific data, Software

## Abstract

There is a generalized consensus in the Non-Intrusive Load Monitoring research community on the importance of public datasets for improving this research field. Still, despite the considerable efforts to release public data, what is currently available suffers from serious issues, among which is the lack of widely accepted data models and common interfaces to access the currently available and future datasets. This paper proposes the Energy Monitoring and Disaggregation Data Format (EMD-DF64). EMD-DF64 is a data model, file format, and application programming interface developed to provide a unique interface to create, manage, and access high-frequency (≥ 1 Hz) electric energy consumption datasets. More precisely, the present paper describes the data model and its respective implementation, which was done by leveraging the well-known Sony WAVE64 format that supports the storage of audio data and metadata annotations.

## Introduction

Non-Intrusive Load Monitoring (NILM), or more generally load/energy disaggregation, is a promising approach for enabling a cost-effective way of providing detailed information about the energy consumption of individual appliances^1^. The availability of public datasets opens new prospects for this technology, helping researchers create more systematic evaluation processes that can be used across different approaches, similar to what happened in other application domains of machine learning like face and speech recognition^2^.

Nevertheless, despite the tremendous efforts in releasing public datasets as reviewed in^3,4^, not many steps have been taken towards homogenizing the way these are made available and accessible to the community. In fact, Several data formats have been employed to store NILM datasets, with Comma-separated values (CSV) emerging has one of the most used formats given the simplicity of the data representation, e.g.,^5–7^. Yet, many other formats were adopted, namely HDF5^8^, relational databases^9^, as well as audio/video formats such as FLAC^10,11^, WAVE^12,13^, and Matroska Media Containers^14^. A direct consequence of the variety of data formats is that in order to use a dataset, researchers first have to understand the underlying structure of the data and produce code to interface with their algorithms. In fact, in^15^ the authors have identified the lack of convergence regarding a storage file format as one of the main challenges to NILM dataset interoperability.

Against this background, some research efforts have been devoted to homogenize existing datasets and provide a single interface to run evaluations. Two examples of such efforts are the Metadata proposal for NILM datasets^16^, and the Non-Intrusive Load Monitoring Toolkit (NILMTK)^17^. The two projects have been combined to form the NILMTK-DF, the data format supported by NILMTK that relies on HDF5, and the metadata structure proposed in^16^. Another example is the NilmDB project^18^, which provides a generalized user interface to access, query, and analyze large time-series datasets, including NILM data. The underlying timestamped data is organized hierarchically and must follow a data stream layout (the number of columns and corresponding data type) determined when a data stream is created. Besides the time series data, NilmDB also supports attaching metadata in the form of key/value pairs.

In^19^ we proposed the Energy Monitoring and Disaggregation Data Format (EMD-DF), a standard data model and file format developed with the intent to represent and store high-frequency datasets (i.e., sampling rate greater or equal to 1 Hz). In EMD-DF, the datasets are represented using the well-known Waveform Audio File Format (WAVE) (see http://fileformats.archiveteam.org/wiki/WAV). The idea of using an audio-based format to represent electric energy data was inspired by previous works where sound-cards are used to perform the acquisition of the current and voltage signals^10,20^, and have been shown to perform considerably well given the similarities to electricity waveforms^21^. Furthermore, being an extension of the Resource Interchange File Format (RIFF) (see http://fileformats.archiveteam.org/wiki/RIFF), the WAVE format has several properties that are desirable in the context of NILM datasets. More particularly:

1. The consumption data, ground-truth, and metadata are all stored in the same file, thus limiting the number of artifacts to be managed;
2. The resulting files are optimized to have very little overhead. Furthermore, since the sampling rate is fixed, only the initial timestamp is necessary to obtain the time of the remaining samples;
3. It is an uncompressed lossless format, i.e., all the original values of the data are kept untouched. Furthermore, it is fully compatible with audio-compression libraries such as WavPack (see https://www.wavpack.com/);
4. It is possible to extend the format at any time with additional chunks without breaking the file consistency, i.e., a WAVE file with additional chunks will consistently be recognized as a WAVE file. Hence backward compatibility with previously developed applications is guaranteed;
5. Finally, there is a diversity of mature programming interfaces to work with audio content, thus facilitating dataset manipulation and portability across different programming environments.

However, extending the WAVE file format comes with two major limitations:

1. To avoid having to store individual timestamps for each sample, the data must not contain missing values. Therefore, missing data is handled separately, either by: (i) resampling whenever possible, i.e., when the number of missed samples is short and sparse, (ii) break the datasets in different files when missing big blocks of data, and (iii) resampling and breaking into multiple files when the missed data is both sparse and with large gaps.
2. The WAVE specification uses a 32-bit unsigned integer to represent the file size header. As such, dataset files are limited to a maximum of 4 GB. This is equivalent to roughly 248 days of two 16-bit channels sampled at 50 Hz. Still, there are datasets with sample rates in the order of kHz. For example, BLUED^22^ was sampled at 12 kHz, meaning that each data file can only represent around 16 h of the three 16-bit channels (i.e., two currents phases and one voltage phase).

In this paper, we present a 64-Bit version of EMD-DF (EMD-DF64) by including support to the Sony WAVE64 (see http://fileformats.archiveteam.org/wiki/Sony_Wave64) file format. Ultimately, this leads to a maximum file size of approximately 16 exabytes, which is equivalent to roughly 21 years of three 16-bits channels sampled at 4.3 GHz). Furthermore, EMD-DF64 was developed to support missing data by default, which is achieved by adding additional chunks to represent such gaps.

In the remainder of this paper, we first present a data model that supports EMD-DF64. Then, we present the data structure, which is an extension of the 64 bit Sony Wave64 file container. Then, we thoroughly describe how the data model and data structure are combined to form the EMD-DF64 file format. Finally, we discuss how the proposed data format contributes to the ongoing efforts to homogenized electricity consumption datasets.

## Methods

### Data model

**Figure 1:**
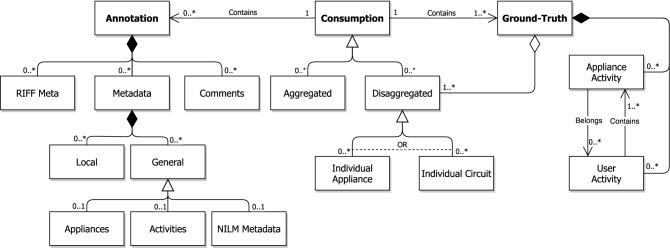
EMD-DF64: Data model overview.

The data model that supports EMD-DF and EMD-DF64 is comprised of several data entities that should be present in a dataset to make it suitable for NILM research. Figure 1 shows an illustration of the proposed data model using the Unified Modeling Language (UML) (see https://www.uml.org/) notation. Overall, there are three main data entities: (1) *consumption*, (2) *ground-truth*, and (3) *annotations*. These are described next.

The *consumption* entity represents all the data elements that refer to energy consumption time-series. Consumption data can be of two different types: (i) *raw waveforms*, i.e., current and voltage; or (ii) *processed waveforms*, i.e., different metrics like real and reactive power. Furthermore, consumption data can represent both *aggregated* and *disaggregated* consumption. Finally, the latter can represent the consumption of individual appliances (e.g., a kettle) or an individual circuit (e.g., kitchen outlets that may or not contain the kettle). It is important to remark that all the entities that refer to consumption data are optional (cardinality of 0 or more) to cover as many variations of NILM datasets as possible. For example, BLUED contains only aggregated consumption data, while SustDataED^13^ contains aggregated and individual appliance consumption. Likewise, RAE^23^ contains data for aggregated, individual appliances and individual circuit consumption. Finally, it is also possible to find datasets that do not contain any form of aggregated consumption, e.g., PLAID^24^ , Tracebase^25^ and GREEND^26^.

On the right-hand side of Fig. 1 is the *ground-truth* entity. This entity is mandatory on a NILM dataset, and can be of four different types: (i) *individual appliance consumption*, (ii) *individual circuit consumption*, (iii) *appliance activity*, and (iv) *user activity*. Individual appliance and individual circuit consumption are a special type of consumption data used to train, test, and validate event-less approaches. On the other hand, appliance activities provide information about the power events that exist on a dataset and are required to train, validate, and test event-based approaches.

We have also introduced the concept of user activities, which is straightforward terms refer to actions that people perform involving the use of electric appliances, e.g., doing the laundry (involves clothes washer, clothes dryer, and iron) or preparing a meal (oven, stove, microwave, aid choppers, and blenders). Such user activities are important to evaluate different NILM application domains, e.g., Non-Intrusive Activity Detection (NIAD)^27^, and the detection of abnormal consumption behaviors^28^. It is important to note that one individual appliance activity can only be associated with one user activity (cardinality of 0 or 1). Otherwise, the total consumption of the user activities will be larger than the total consumption of the individual appliances, thus introducing inconsistency to the data model.

Lastly, we have the *annotations* entity. There are three types of optional annotations: (1) *RIFF Meta*, which are the default metadata chunks defined by RIFF, (2) *comments*, consisting of free text, and 3) user-defined *metadata*. The latter can be either which can be either *local metadata*, which refers to specific samples in the consumption data, or *custom metadata* annotations defined by the dataset creator and can serve multiple purposes. For example, in the current implementation, it is possible to provide a list with details for individual appliances and user activities and embed the annotations from the NILM metadata project.

### Data structure

In EMD-DF64, the different data entities are represented by extending the 64-bit Sony WAVE64 (SW64) file format. SW64 is an application of the RIFF in which the file contents are grouped and stored in separate chunks. Each chunk consists of three components: (1) chunk identifier—128-bit globally unique identifier (GUID), e.g., “fmt” and “data”; (2) an unsigned, little-endian 64-bit integer representing the length of the chunk; (3) the chunk data. Finally, like all other RIFF-based formats, if a chunk’s data size is not even, it is padded by 1 byte to make it so.

A WAVE64 file is composed of several chunks, four of which are mandatory. Furthermore, in a correctly formatted WAVE64 file, the first four bytes (GUID) must always spell out “riff” (lower-case). Some of the W64 chunks are briefly described in Table 1, with a particular focus on those reused in the EMD-DF data model. For more details about the RIFF standard, please refer to the original project documentation in^29^.

**Table 1:** List of chunks that compose the RIFF-WAVE64 file format.

Name	GUID	Description	Parent	EMD-DF64
RIFF^a^	‘riff’	This is the main chunk and is mandatory for every file that is based on the RIFF standard	–	$\checkmark$
WAVE^a,b^	‘wave’	Identifies the contents of the RIFF chunk as being of the type w64	riff	$\checkmark$
Format^a^	‘fmt ’	Defines the data format, e.g. sampling rate, sample size and bits and the number of channels	riff	$\checkmark$
Data^a^	‘data’	Waveform data can be stored as a single contiguous array of interleaved samples or as a discrete sequence of blocks of samples and silence wrapped in a ‘wavl’ chunk	riff	$\checkmark$
Wave list	‘wavl’	Wraps sequences of data and silence chunks	data	–
Silent	‘slnt’	Represents silence and is defined as a count of silence samples	wavl	–
Fact	‘fact’	Stores information about how the waveform data is organized. It is mandatory when the waveform data is stored in a ‘wavl’ chunk and for all compressed audio formats	riff	–
Cue	‘cue ’	Identifies a series of positions in the waveform data as as having additional information associated with them. There is at most one cue chunk per wave file, and it is followed by a list of cue points	riff	$\checkmark$
List	‘list’	This is a wrapper for RIFF chunks, which in the particular case of WAVE64 files is an associated data list (“adtl”)	riff	$\checkmark$
Associated data list^a,b^	‘adtl’	Identifies a list that contains individual information attached to the cue points defined in the cue chunk	list	$\checkmark$
Label	‘labl’	Associates a text label to a specific cue point. Must be defined inside the associated data list chunk	adtl	$\checkmark$
Note	‘note’	Same as label, but usually contains comment text for a specific cue point	adtl.	$\checkmark$
Labeled text	‘ltxt’	Associates a text comment to specific regions of waveform data. A region is a cue point whose adtl list duration in samples is defined in this chunk. Must be defined inside the associated data list chunk	adtl	$\checkmark$
Embedded file info	‘file’	Contains information described in other file formats (e.g. ASCII text files) that is associated with a particular cue point	adtl	–
Playlist	‘plst’	Specifies a play order for a series of cue points	riff	–
Info^b^		Identifies a list that contains the info chunks^29^	riff	$\checkmark$

^a^ These chunks are mandatory.

^b^‘WAVE’ , ‘adtl and ‘INFO’ are chunk identifiers.

**Figure 2:**
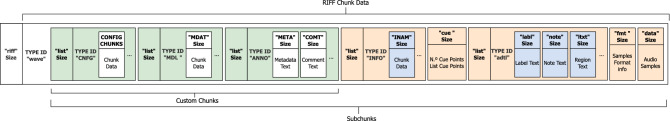
Chunk structure of the EMD-DF 64 file format.

The EMD-DF64 file format is based on 20 chunks. One directly inherited from the RIFF standard (*Info*), nine from the WAVE64 format (*Format*, *Data*, *Cue*, *List*, *Associated Data List*, *Label*, *Note* and *Labeled Text Chunk*), and the remaining 10 are custom chunks. The chunk structure of EMD-DF64 is illustrated in Fig. 2. Table 2 provides a description of the 10 custom chunks.

**Table 2:** List of EMD-DF64 specific chunks.

Name	GUID	Description	Parent
Config^a^	“CNFG”	List with file specific configuration chunks	riff
Timestamp^a^	“TMSP”	Unix timestamp of the first sample in the waveform data	CNFG
Timezone^a^	“TMZN”	Timezone of the place where the data was collected	CNFG
Sampling rate^a^	“SPRT”	Sampling rate of the waveform data (overwrites the original value in the format chunk if the actual sampling rate is lower than 1 Hz)	CNFG
Calibration constants^a^	“CHCC”	Calibration constants to recreate the original values of the waveform data. One constant for each channel	CNFG
Missing data list	“MDL”	List with missing data identifiers	riff
Missing data	“MDAT”	Chunk containing a missing data identifier	MDL
Annotation	“ANNO”	Identifies a list that contains metadata and comment chunks	riff
Metadata	“META”’	This is metadata specific chunk. Must be contained in the Annotation chunk	ANNO
Comment	“COMT”	This is a comment specific chunk and must be specified within the Annotation chunk	ANNO

^a^These chunks are mandatory.

### EMD-DF64 file format definition

Next, we present how the different chunks that compose the EMD-DF64 data structure are combined to create a dataset. We first describe how the data format is defined in the Format and Config chunks. Then we show how the power measurements are stored and supplemented with the different embedded annotations.

#### Waveform data format

The waveform data (i.e., consumption data) must be defined in the *Format* chunk. This is inherited from the W64 format and consists of the following fields: (i) *sample size* in bits (8, 16, 24, 32 or 64 bits); and (ii) *number of individual channels* (greater or equal to 1).

Additionally, all the sub-chunks defined in the *Config* list chunk are mandatory. More precisely: (i) *timezone* (the time zone of the location where the data was collected), (ii) *timestamp* (the Unix timestamp of the first sample in the waveform data), (iii) *sampling rate* (the number of samples per second in the waveform data), and (iv) *calibration constants* (zero or one for each waveform channel). The calibration constant chunks are associated to each channel in ascending order. For the model to be valid, the number of calibration chunks must be zero (i.e., no calibration is needed) or equal to the number of individual channels.

#### Consumption data

The consumption data are represented using the *Data*, *Missing Data List*, and *Missing Data* chunks. The *Missing Data List* wraps sequences of *Missing Data* chunks to support datasets with missing data.

Waveform data are stored uncompressed in *Data* chunks. If only one metric needs to be represented (this is the case in most individual appliance and circuit ground-truth data), the samples are stored consecutively; otherwise the samples are stored interleaved. Each sample *S* is represented by a value between − 1 and 1. Samples are stored in little-endian format (i.e., the least significant byte is stored first). The bits that represent the sample amplitude are stored in the most significant bits of *S*, and the remaining bits are set to zero.

##### Handling missing data

Intervals with missing data are represented using the *Missing Data* chunk. Each of these chunks contains a JSON string with information about the timestamp when data is again available, and the number of the sample where this happens.
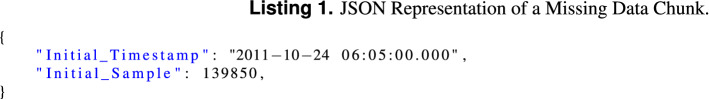


##### Timestamp conversion

Since we do not store the timestamp of each individual sample, this has to be calculated in run-time. This is done using Eq. (1), which returns a Unix timestamp in milliseconds:

1 $$\begin{aligned} unix\_timestamp = 1000 \times \frac{current\_sample - initial\_sample}{f} + initial\_unix\_timestamp \end{aligned}$$

Where *current_sample* is the position of the sample of interest, *initial_sample* is the position of the first sample (if there are no missing data chunks the first sample is 1, otherwise it is the initial sample of the corresponding missing data chunk). *initial_unix_timestamp* is the unix timestamp of the first sample (if there are no missing data it is given by the Timezone chunk, otherwise it is calculated from the corresponding missing data chunk). Finally, *f* is the sampling rate of the waveform data.

Conversely, it is possible to convert a unix timestamp to a sample position. This is done using Eq. (2):

2 $$\begin{aligned} position = \frac{actual\_timestamp - initial\_timestamp}{\frac{1}{f} \times 1000} \end{aligned}$$

Where *actual_timestamp* is the timestamp in milliseconds to be mapped to an audio position, *initial_timestamp* is the timestamp in milliseconds of the first sample in the dataset, and *f* is the sampling rate of the waveform data.

#### Ground-truth: individual appliance activity

Individual appliance activities correspond to the changes in the power consumption that are triggered by different appliance turning ON, OFF, or changing their working mode (e.g., low to high).

Individual appliance activity (i.e., power events), are embedded in the file using the *Cue*, *Associated Data List* and *Label* chunks. This is done as follows: (1) For each power event, an entry is added in the Cue chunk, (2) for each Cue chunk entry, a Label chunk is created and added to the Associated Data List chunk.

Each label chunk consists of a sample position in the waveform data and a JSON formatted string with the details of the respective activity. The sample position is calculated from the power event timestamp using Eq. (2): For example, the JSON in Listing 2 corresponds to a refrigerator activity that was mapped to position 19394633:
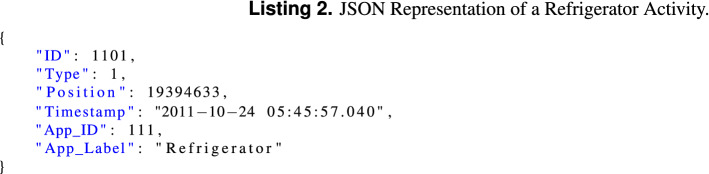


#### Ground-truth: user activity

User activities refer to actions that are performed involving the use of electric appliances, e.g., doing the laundry (involves clothes washer, clothes dryer and iron) or preparing a meal (oven, stove, microwave, aid choppers, blenders, etc.).

User activities are supplemented in the consumption data using the *Cue*, *Associated Data List* and *Labeled Text chunks*. This is done as follows: (1) For each user activity, an entry is added in the Cue chunk, (2) for each Cue chunk entry, a Labeled text chunk is created and added to the Associated Data List chunk.

Each labeled text chunk consists of a sample position in the waveform data, a duration in samples, and a JSON formatted string with the details of the respective activity. Listing 3 shows a JSON representing the “working on the computer” activity that involves using the desktop computer (App_ID: 1101), one monitor (App_ID: 1109) and a printer (App_ID: 1203). The duration in samples is obtained by subtracting the start from the end position of the activity. The sample positions are calculated from the timestamps using Eq. (2).
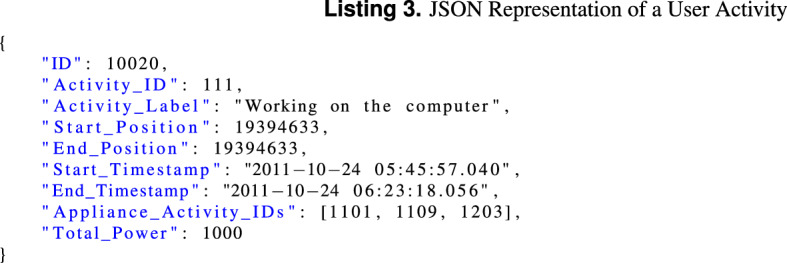


#### RIFF meta

Since EMD-DF64 is a direct application of the RIFF standard, it fully supports all the default RIFF metadata sub-chunks that are defined in the *Info* chunk. These are used to supplement general annotations, and include among others, *Creator*, *Commissioner*, *Copyright*, and *Keywords*. The RIFF metadata chunks supported in EMD-DF64 are listed in Table 3.

**Table 3:** List of RIFF metadata chunks supported by EMD-DF64.

Name	GUID	Description
File creator	“IART”	The name of the file creator
Commissioner	“IMCS”	The name of the dataset commissioner
Comments	“ICMT”	Free text comment
Copyright	“ICOP”	Dataset copyright notice
Creation data	“ICRD”	Data of dataset creation
Keywords	“IKEY”	A list of keywords to describe the dataset content
Name	“INAM”	The dataset name
Product	“IPRD”	Original purpose of the dataset
Subject	“ISBJ”	Contents of the file (e.g., current and voltage waveforms)
Software	“ISFT”	Name of the software that was used to create the file

#### Local metadata

Local metadata annotations can be used for instance to supplement datasets with details like the instant when a new appliance is added or removed from the electric circuit. Local metadata are used to supplement specific consumption samples with custom annotations. For instance, when a new appliance is added or removed from the electric circuit as shown in JSON Listing 4.

A note chunk consists of a sample position and a JSON formatted string. Notes are created using the *Cue*, *Additional Data List* and *Note* chunks as follows: (1) For each local annotation, an entry is added to the Cue chunk, (2) for each entry in the Cue chunk, a Note chunk is added to the Associated Data List chunk.
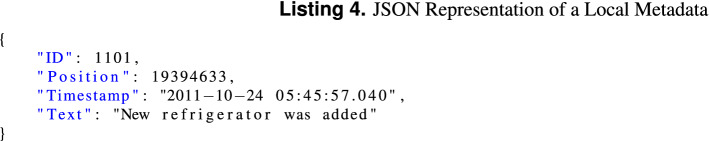


#### General metadata

These custom chunks can be used to enrich datasets with custom metadata. They are added using the *Annotation List* and *Metadata* chunks.

The content of such chunks do not follow any specific rule, yet it must be encoded in JSON and always include the ID and Label fields. EMD-DF64 fully supports three different custom metadata types: (i) *appliances*; (ii) *user activities*; and (iii) *NILM metadata* project annotations.

##### Appliances metadata

Keeps a list of the appliances that co-exist in the dataset, including the appliance characteristics like brand, model, energy consumption and energy efficiency rating. Listing 5 shows a possible JSON representation of the appliances metadata chunk.


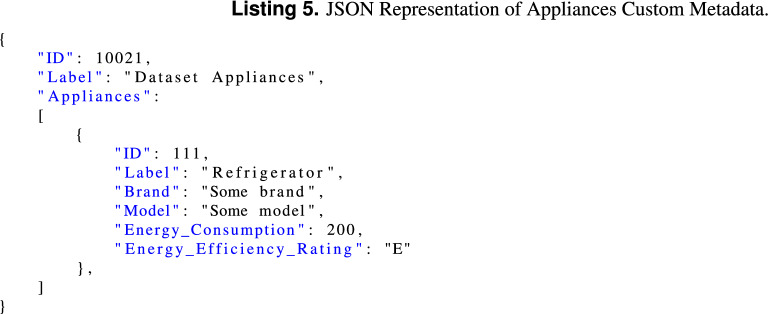


##### User activities metadata

Keeps a list of the user activities that are present in the dataset, including a list of the appliances that can be associated with each activity. An example is provided in Listing 6.


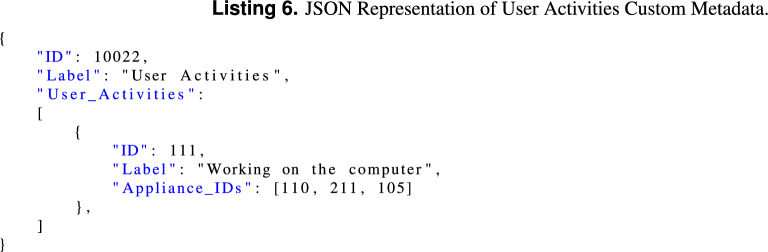


##### NILM metadata

It is also possible to supplement datasets with annotations from the NILM metadata project. To this end we have defined the NILM Metadata Project annotation that can be used to embed the content of the different YAML files that compose the NILM Metadata project, in a metadata chunk. An example annotation is provided in Listing 7.


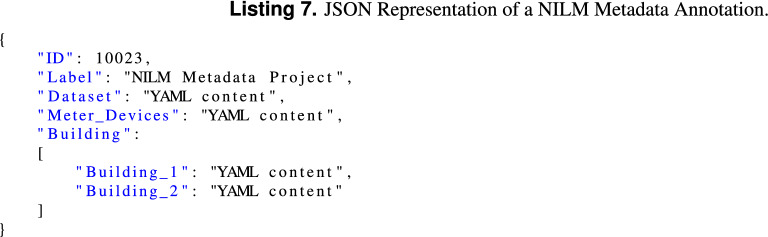


#### Comments

Custom comment chunks consist of free form text and are created using the *Annotation List* and *Comment* chunks. These can be used to add any kind of comments, for example, add a comment containing the historic of previous performance evaluations results on that particular file or dataset. Another example would be, adding a comment regarding some external event that could have affected the data.

#### JSON schemas

Since most of the annotation data will be done using the JSON format we have decided to use JSON schemas (see http://json-schema.org/) to describe the JSON data elements presented in the previous sub-section. Listing 8 shows a snippet of the JSON-Schema for the appliance activity labels.
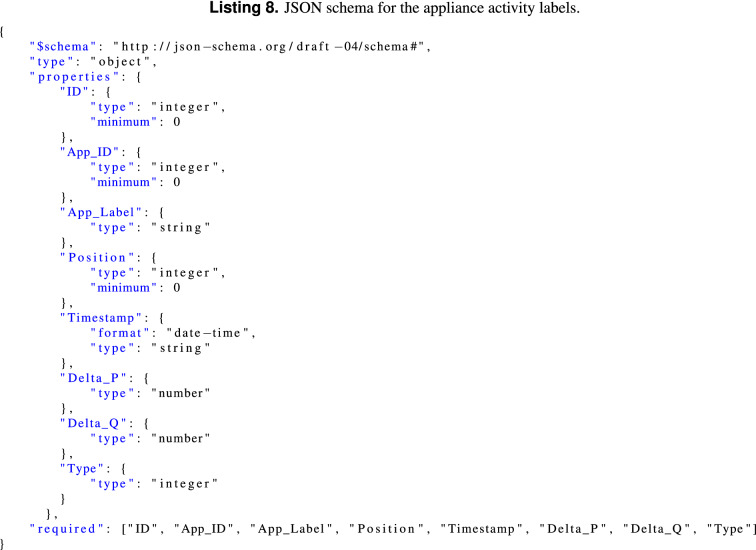


## Implementation and demonstration

The present version of EMD-DF64 was implemented using the Java programming language. This is an open-source project that can be found in an online repository (see https://gitlab.com/alspereira/EMD-DF). Online documentation is also available (see https://manelpereira.gitlab.io/emd-df-documentation/).

**Figure 3:**
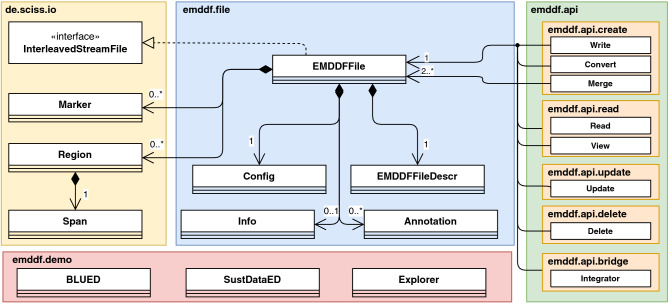
EMD-DF64 class diagram.

The UML class diagram of EMD-DF64 is provided in Fig. 3. The library is composed of three main packages: de.sciss.io, emddf.file, and emddf.api.

The de.sciss.io package is responsible for the audio file I/O and provides also the core classes to add ground-truth annotations (Marker and Region). This package was reused from the ScissLib project (see https://github.com/Sciss/ScissLib).

The emddf.file package provides all the basic structures to represent EMD-DF and EMD-DF64 files. The EMDDFAudioFiles allows reading and writing the audio files. The supported formats are Waveform Audio File Format (WAVE) and Sony Wave64 (W64), which support the EMD-DF, and EMD-DF64 formats, respectively. The EMDDFFileDescr class provides the data structure that describes the format of an EMDDFAudioFile. These two classes were adapted from the ScissLib project classes AudioFile and AudioFileDescr. The remaining classes provide the basic structure to define the different EMD-DF and EMD-DF64 chunks.

The emddf.api package provides an API to manage EMD-DF and EMD-DF64 files. The API was developed around the four basic operations of persistent storage: Create; Read; Update; Delete (CRUD). The API also offers a brige to map the EMDDF-64 behavior in different programming languages.

Finally, the emddf.demo package provides concrete application examples of this EMD-DF64 implementation. Presently there are three demos: i) Explorer that provides a command-line interface to explore different EMD-DF64 datasets, ii) BLUED that shows a concrete example of adding ground-truth labels to an EMD-DF6 dataset, and iii) SustDataED that provides a concrete example of handing missing data in EMD-DF64 datasets. These three demos are brifly described in Table 4.

### Support to additional programming languages

All the modern programming languages (e.g., Python, C++, and MATLAB) have built-in mechanisms to handle audio files. To make the integration with other programming languages easier, a Java Bridge was developed, to expose the functionalities of EMDDF-64. To exemplify its usage, two demos were were developed and released together with EMDDF-64. The first demo uses Python, leveraging the pyemddf (see https://pypi.org/project/pyemddf/) package. The second demo, was developed for MATLAB and leverages the External Language Interfaces (see https://www.mathworks.com/help/matlab/using-java-libraries-in-matlab.html). The two demos are briefly described in Table 4.

**Table 4:** Description of the demos provided along the the EMD-DF64 software package.

Demo	Objective
Explorer	Demonstrates different features of the EMD-DF64. The features include: (1) reading an existing file, (2) writing a new file, (3) updating an existing file, (4) converting between file formats, and (5) merging files.
BLUED	Demonstrates the conversion of the BLUED dataset. This demo includes adding appliance labels, appliance activities, and metadata (local notes, comments, and RIFF Info chunks)
SustDataED	Demonstrates the conversion of the SustDataED dataset. This demo includes adding labels to datasets with missing data, as well as merging dataset files
Python_demo	Python notebook that demonstrates the application of the pyemddf to handle EMD-DF64 files in Python
MATLAB_demo	MATLAB script that demonstrates the handling of EMD-DF64 files using the EMDDF.m class

### Comparison with other formats

As mentioned in the introduction, one of the key advantages of using an audio format to store high-frequency datasets is the overall file size reduction. Therefore, this section presents a comparative analysis of three audio formats, namely, Free Lossless Audio Codec (FLAC), WAVE, and W64. FLAC was introduced in^10^ to store the UK-DALE, whereas WAVE and W64 are the two formats that support EMD-DF and EMD-DF64, respectively.

The comparisons are performed using the Fully Labeled Public Dataset for Event-Based Non-Intrusive Load Monitoring Research (BLUED), considering the raw current and voltage waveforms. The original BLUED distribution contains one week of energy consumption of a two-phase electric system from a household in the United States. The current and voltage signals were sampled at 12 kHz, and are available in text files (.txt) containing a timestamp and the values for current (phase A and B), and voltage (phase A). Before conversion, the raw current and voltage waveforms were scaled to − 1 and 1 by dividing each sample by the maximum value of each signal (75 and 180, respectively). Then, the scaled waveforms were converted to FLAC, WAVE, and W64 using the dsCleaner library^30^. The following format parameters were used: sampling rate: 12 kHz, sample size: 16 bits and number of channels: 2 (current and voltage). Individual files were created for each phase, by assuming that the voltage of phase B is the voltage of phase A shifted by $90^{\circ }$.

For comparison, the following quantities were considered: (1) the number of files based on the maximum file size; (2) the total size (in GB), (3) compressed size using WavPack compression. The results are summarized in Table 5.

**Table 5:** Comparison of file formats using BLUED as a reference dataset.

	TXT	FLAC	EMD-DF (WAVE)	EMD-DF64 (W64)
Current and voltage files	6430	32	14	2
Total size	320^a^	27	55	51
WavPack compress (GB)	–^b^	–^a^	30	28

^a^54 GB when compressed using RAR (see https://www.rarlab.com/).

^b^Not applicable.

As expected, the three audio formats enable a very significant decrease in the dataset size without losing any information. In the case of FLAC the reduction is around 92%, whereas for WAVE and W64 the reduction is 82% and 85%, respectively. However, it should be stressed that FLAC is at its core an audio compression tool, hence the considerably lower size of the resulting files. Therefore, to achieve a fair comparison, the WavPack audio compressor tool was used to compress the WAVE and W64 files. Ultimately, this resulted in a reduction in the file size of 90%, and 91% for WAVE, and W64, respectively.

Finally, regarding the number of files, it was possible to represent the entire dataset in two files (one per phase) when using the W64 format. As for the WAVE format, due to the limitations of the 32-bits header, it was necessary to break the dataset into 7 files for each phase. Finally, in the case ofFLAC, it was not possible to merge the files using the dsClearner API since read/write operations are not allowed in the FLAC format. Instead, external software, e.g., Audacity (see https://www.audacityteam.org/), would be required to perform the merge operation manually.

## Discussion

EMD-DF64 was developed to help mitigate the lack of homogeneity across existing electricity consumption datasets, which poses significant challenges for researchers intending to use datasets comparatively.

In^15^, the authors offered 17 suggestions for improving the collection, storage, and provision of electricity datasets. Next, we briefly discuss how EMD-DF64 promotes adopting some of those suggestions from a data storage and representation point of view.

*Suggestion 5: gaps and irregularities* EMD-DF64 supports missing data by default. Furthermore, by indicating the timestamps with missing data, it is straightforward to implement data cleaning strategies such as data interpolation. Local metadata can also be used to annotate regions where data is out of distribution (e.g., considerable changes to the voltage signal).

*Suggestion 10: annotation of traces of event/activity information* EMD-DF64 supports embedded annotations for both appliances (labels) and user activities (labeled text) by default. Furthermore, local metadata (notes) can be used to embed information on relevant changes, e.g., addition/removal/replacement of an appliance or changes to the number of household members.

*Suggestion 12: metadata formatted in reusable machine way/easy processing* In EMD-DF64, metadata is represented using JSON, a widely used data-interchange format. Besides that, it is lightweight (e.g., compared to XML) and easy for humans to read and write. Furthermore, to remove ambiguity and errors when labeling the dataset, the annotations are validated using JSON schemas before being embedded in the data files.

*Suggestion 14: compatibility of formats with software and compression* By extending an audio format, it is possible to take advantage of the many audio libraries available in the different programming languages. For instance, in our particular case, we provide a Java implementation of EMD-DF64, which can be easily integrated with Python using the pyemddf package.

As for compression, EMD-DF64 is fully compatible with the WavPack audio compression format, which provides both lossless and hybrid (i.e., lossy with the possibility of complete restoration) compression modes. The compression ratio depends on the source material but generally is between 30% and 70%. For example, using SustDataED, we have observed compression rates of 52% for raw current and voltage waveforms sampled at 12.8 kHz. On the other hand, a rate of 70% was achieved for active power, reactive power, current, and voltage measurements sampled at 50 Hz.

## Conclusion

This paper presented a 64-bits file format for representing and storing high-frequency energy monitoring and disaggregation datasets. While we are fully aware that another format alone is not enough to solve the problem of lack of convergence between file formats in NILM research, we believe that in the era of big data, datasets will not only grow in volume but also in variety. Thus, it is necessary to provide mechanisms that are already optimized to handle such high volumes of data while also supporting a wide variety of mechanisms for metadata annotations, which is the case of audio file formats.

While different file formats have been proposed in the past, as summarized^15^, one of the main challenges is to increase the adoption by the research community. To this end, in immediate future work, we will integrate EMD-DF64 datasets in the Non-Intrusive Load Monitoring Toolkit (NILMTK) pipeline. This will not only increase the visibility of EMD-DF64 but also mitigate one of the current limitations of NILMTK which is the fact that the underlying NILMTK Data Format (NILMTK-DF) is not adequate to handle high-frequency datasets.

One of the limitations of EMD-DF64 is the fact that it does not support dataset querying by default, which is supported in alternative formats like relational databases and Hierarchical Data Format (HDF5). Therefore, another important future research direction would be to extend the API module with query operations. For example, loading the consumption waveforms that are associated with a given user activity. Furthermore, this query language should also allow the integration between aggregated and individual appliance consumption, which lies in different EMD-DF64 files.

Finally, it should be noted that although the current work deals with electricity consumption datasets, all the underlying concepts can be extended to support other types of time-series data. Moreover, since most of the annotations are added using JSON strings, any adaptation to other types of datasets should be very straightforward.

## Data Availability

All the data necessary to run the demo applications (BLUED and SustDataED) are available in the projects’ OSF repository^31^. All the datasets used in the demo applications are available from the corresponding author on reasonable request. Please refer to the original publications for additional details.

## Code availability

The present version of EMD-DF64 was implemented using the Java programming language. The EMD-DF64 implementation is released under the GNU General Public License (GPL) 3.0. Source-code and documentation are available in the project page at the Open Science Framework (OSF)^31^.
